# The accuracy of ultrasound-guided fine-needle aspiration and core needle biopsy in diagnosing axillary lymph nodes in women with breast cancer: a systematic review and meta-analysis

**DOI:** 10.3389/fonc.2023.1166035

**Published:** 2023-06-21

**Authors:** Haining Zheng, Rui Zhao, Wei Wang, Xiaona Liu, Xiaoqing Wang, Chaoyang Wen, Yubo Ren

**Affiliations:** ^1^ Dpartment of Ultrasound, Peking University International Hospital, Beijing, China; ^2^ Department of Ultrasound, Fourth Medical Center of Chinese People's Liberation Army General Hospital, Beijing, China; ^3^ Department of Ultrasound, Beijing Hospital of Traditional Chinese Medicine, Beijing, China; ^4^ Department of Pathology, Peking University International Hospital, Beijing, China

**Keywords:** meta-analysis, fine-needle aspiration, core needle biopsy, axillary lymph nodes, diagnostic accuracy

## Abstract

**Background:**

This study evaluates the diagnostic accuracy of ultrasound-guided fine needle aspiration (US-FNA) and core needle biopsy (US-CNB) for detecting axillary lymph nodes in women with breast cancer.

**Methods:**

Eligible studies and pertinent literature resources were identified in Cochrane, PubMed, Embase, CNKI, VIP, and Wanfang databases using subject-specific keywords. Study outcomes were tested for heterogeneity, and meta-analyses were performed to estimate sensitivity, specificity, and diagnostic odds ratios (DORs). The summary receiver operating characteristic (SROC) curve analysis was also performed.

**Results:**

A total of 22 studies involving 3,548 patients were included to evaluate the diagnostic accuracy of US-FNA and 11 studies involving 758 patients were included to evaluate the diagnostic accuracy of US-CNB in identifying axillary lymph nodes in women with breast cancer. The accuracy of US-FNA in identifying suspicious axillary lymph nodes was as follows: overall sensitivity, 79% (95% CI: 73%–84%); global specificity, 96% (95% CI: 92%–98%); overall positive likelihood ratio, 18.55 (95% CI: 10.53–32.69); overall negative likelihood ratio, 0.22 (95% CI: 0.17–0.28); DOR, 71.68 (95% CI: 37.19–138.12); and the area under the SROC curve, 0.94 (95% CI: 0.92–0.96). The accuracy of US-CNB in identifying suspicious axillary lymph nodes was as follows: overall sensitivity, 85% (95% CI: 81%–89%); global specificity, 93% (95% CI: 87%–96%); overall positive likelihood ratio, 11.88 (95% CI: 6.56–21.50); overall negative likelihood ratio, 0.16 (95% CI: 0.12–0.21); overall DOR, 66.83 (95% CI: 33.28–134.21), and the area under SROC curve 0.96 (95% CI: 0.94–0.97).

**Conclusions:**

The results indicate that both US-FNA and US-CNB have high accuracy for suspicious axillary lymph nodes.

## 1 Introduction

Axillary lymph node (ALN) metastasis is an important factor in the clinical evaluation of the prognosis of breast cancer. Conventional ALN dissection (ALND) plays a critical role in the staging of breast cancer, but it is associated with serious postoperative complications that affect postoperative recovery. Sentinel lymph node biopsy (SLNB) is also used for the assessment of the ALN stage and the formulation of planning of treatment. However, SLNB requires accurate preoperative positioning and pathological diagnosis results, which is also prone to false-negative results. Therefore, developing a simple and effective diagnostic method is of pivotal importance. Imaging examination as a non-invasive examination method is frequently applied in clinical diagnosis and treatment and can help to effectively diagnose ALN and avoid unnecessary SLNB (1–4).

Ultrasound is a commonly used imaging method to evaluate the properties of ALN. With its real-time dynamics, simple operation, and non-invasiveness, ultrasound can explore ALN from multiple angles and directions. In recent years, ultrasound-guided needle biopsy, contrast-enhanced ultrasound, and elastography have enriched the ultrasonic diagnosis of ALN. Compared with conventional ultrasound, ultrasound-guided percutaneous biopsy results are more accurate and effective in the diagnosis of ALN metastasis (5–8). By identifying relevant studies from scientific literature, the present study aimed to evaluate the accuracy of ultrasound-guided fine needle aspiration (US-FNA) and core needle biopsy (US-CNB) in detecting suspicious ALNs in women with breast cancer.

## 2 Methods

### 2.1 Literature search

All the eligible studies analyzing the diagnostic accuracy of US-FNA and US-CNB in detecting ALNs in women with breast cancer were searched in Cochrane, PubMed, Embase, CNKI, VIP, and Wanfang databases. Other related correlational studies or referenced data were also retrieved. Two researchers independently retrieved the articles, and a third researcher was involved to resolve any disagreements.

### 2.2 Inclusion and exclusion criteria

The criteria for study inclusion were as follows: (1) cohort or cross-sectional research design; (2) evaluated the diagnostic accuracy of suspicious ALNs in women with breast cancer; (3) suspicious ALNs were diagnosed using US-FNA and/or US-CNB; (4) reported true positive (TP), false positive (FP), false negative (FN), and true negative (TN) data; and (5) publication language was English or Chinese. The criteria for exclusion were as follows: (1) duplicate articles, or articles with the same results; (2) case reports, theoretical studies, conference presentations, review of literature, meta-analysis, expert commentary, or analyses; (3) research articles without results relevant to this study; and (4) without clinical outcomes of TP, FP, FN, or TN. Two researchers decided whether the article was to be included with a third researcher helping to resolve any disagreements.

### 2.3 Data extraction and quality evaluation

Extraction of data was performed independently by two researchers with the help of a third researcher who was involved to resolve any disagreement. For the clinical outcomes, 2 × 2 diagnostic table (TP, FP, FN, and TN) data were sought for each of the included articles. Sensitivity, specificity, and likelihood ratios were computed. Accuracy of US-FNA and US-CNB in diagnosis was measured with the diagnostic odds ratio (DOR). When DOR equaled one, it suggested no distinguishing ability, whereas a higher value indicated a higher correlation of the evaluated diagnostic tool.

### 2.4 Statistical analysis

Stata 10.0 (TX, USA) was used for all statistical analyses. Statistical heterogeneity was determined with the chi-squared test and *I* ^2^ values. If the *p*-value of the chi-squared test was equal to or lower than 0.05 and *I* ^2^ was higher than 50%, the random-effects model was chosen for meta-analysis; otherwise, the fixed-effects model was used. Based on the correlation analysis (Spearman’s) between the logarithm of sensitivity and the logarithm of [1 − specificity], the presence or absence of the effect of threshold was checked to further investigate the heterogeneity. In the presence of the threshold effect, there should be a negative correlation between the sensitivity and specificity (or a positive correlation between sensitivity and [1 − specificity]). A strong positive correlation between sensitivity and [1 − specificity] indicates the effect of the threshold. The summary receiver operating characteristic (SROC) curve was analyzed when the heterogeneity was caused by the effect of the threshold. The overestimated overall values of sensitivity and specificity were evaluated by this method. The publication bias was assessed using Deeks’ Funnel Asymmetry Plot.

## 3 Results

### 3.1 Essential features of the included publications

**Figure 1:**
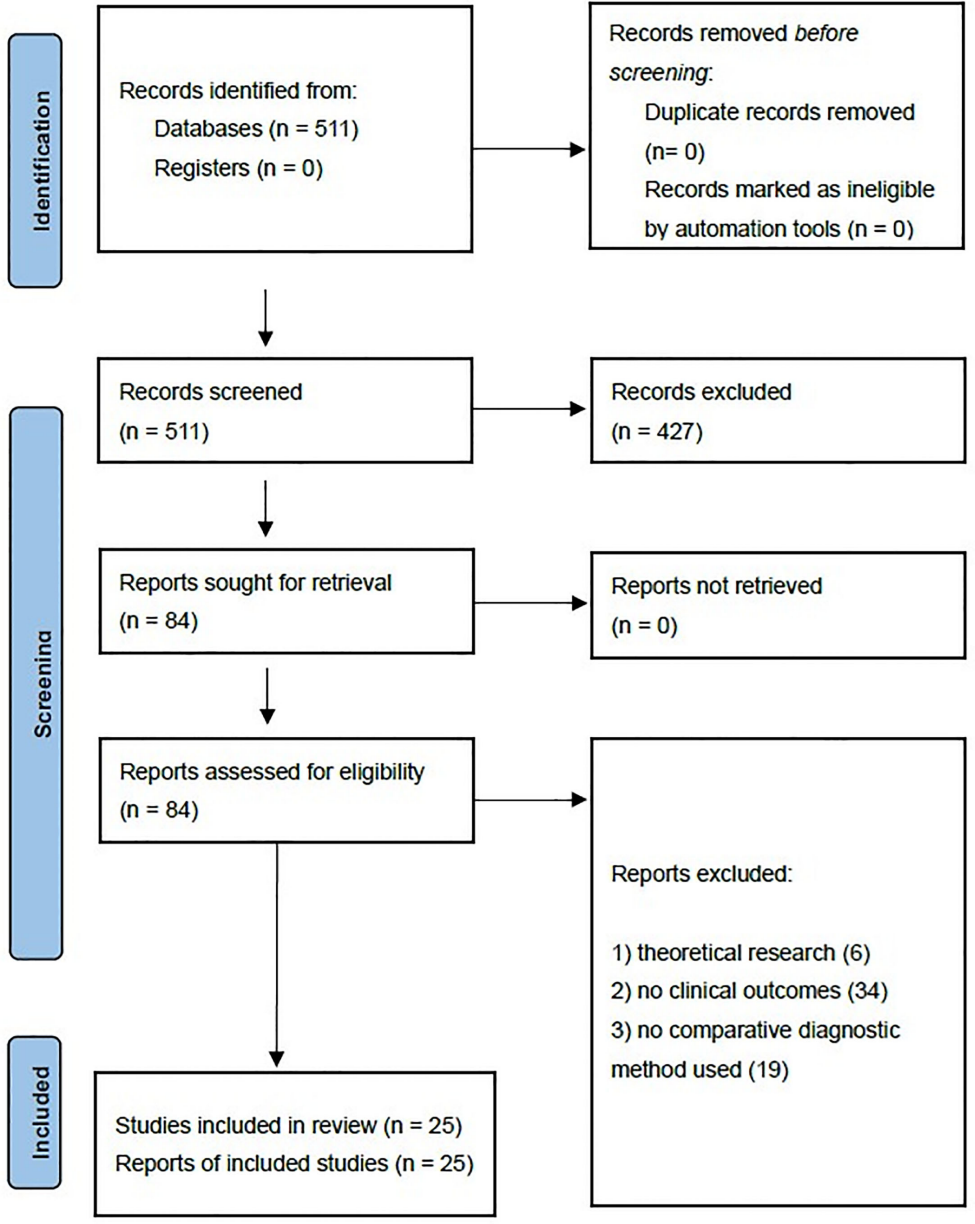
Flow diagram of the literature search and selection process.

A total of 511 publications were identified by searching keywords. After initial screening, 427 publications were excluded after reviewing the title or abstract, and 84 publications were subjected to further assessment. Fifty-nine publications failed to meet the inclusion criteria because they were theoretical research (6), reports without clinical outcomes (9), and studies without comparative diagnostic methods (10). Finally, 25 studies (9–34) with 4,354 patients were included in this meta-analysis. The flow path is shown in **Figure 1**. Important characteristics of these studies are given in **Table 1**.

**Table 1:** The basic characteristics of included studies.

Study	Sample	Age	Diagnostic Method	TP	FP	FN	TN
Rikiya Nakamura 2017 a	487	54	US-FNA	367	1	199	96
Rikiya Nakamura 2017 b	172	56	US-CNB	214	0	30	12
Raghavan Vidya 2017 a	43	–	US-FNA	18	0	7	25
Raghavan Vidya 2017 b	38	–	US-CNB	27	0	0	11
Roshni Rao 2009 a	22	50.5	US-FNA	12	0	4	6
Roshni Rao 2009 b	25	52.5	US-CNB	18	0	4	3
Marie A. Ganott 2014 a	95	–	US-FNA	55	0	10	5
Marie A. Ganott 2014 b	95	–	US-CNB	61	0	4	5
Hye Shin Ahn 2013 a	48	49	US-FNA	19	0	7	22
Hye Shin Ahn 2013 b	48	49	US-CNB	20	0	6	22
Suvi Rautiainen 2013 a	178	61.4	US-FNA	37	0	14	15
Suvi Rautiainen 2013 b	178	61.4	US-CNB	45	0	9	15
A.J. Maxwell 2016	40	57	US-CNB	15	0	4	18
B. J. van Wely 2013	199	–	US-FNA	157	0	19	22
Franco Genta 2007	370	–	US-FNA	43	0	23	31
MB Popli 2006	24	–	US-FNA	15	0	4	5
U˘gur Topal 2005	39	–	US-CNB	30	0	3	6
Savitri Krishnamurthy 2002	103	–	US-FNA	51	16	12	24
Yanbin Wang 2005	86	48.9	US-FNA	50	0	5	31
Yan Ding 2018	148	49.2	US-FNA	64	0	17	75
Shichong Zhou 2017	500	45.2	US-FNA	192	0	54	136
Jian Le 2017	255	52	US-FNA	105	3	24	123
Juan Wang 2013	87	51.2	US-FNA	60	0	4	20
Leijun Huo 2016	89	46.29	US-FNA	19	1	15	8
Ying Sang 2016 a	48	49	US-FNA	19	0	7	22
Ying Sang 2016 b	48	49	US-CNB	20	0	6	22
Chunyang Yu 2018	27	47	US-CNB	35	1	2	3
Guo Sang 2019	58	48.3	US-FNA	37	0	7	14
Yibo Zhao 2015	454	49	US-FNA	25	0	14	61
Miao Liu 2011	40	52	US-FNA	12	0	11	17
Yuntao Wei 2014	47	52.2	US-FNA	24	5	0	18
Yajun Ruan 2018	167	53.2	US-FNA	73	1	2	20

US-FNA, Ultrasound-guided fine-needle aspiration; US-CNB, ultrasound-guided core needle biopsy; TP, true positive; FP, false positive; FN, false negative; TN, true negative.

a: patients diagnosed by US-FNA, b: patients diagnosed by US-CNB.

### 3.2 Diagnostic accuracy of US-FNA

A total of 22 studies with 3,548 patients were included to estimate the diagnostic accuracy of US-FNA in identifying ALNs in women with breast cancer. The random-effects model was selected for meta-analysis based on the presence of high heterogeneity (*p* < 0.001, *I* ^2 = ^59.2%). The correlation result (Spearman’s *R* = −0.2331, *p* = 0.2965) between the logarithm of sensitivity and the logarithm of [1 − specificity] indicated that there was no effect of the threshold.

**Figure 2:**
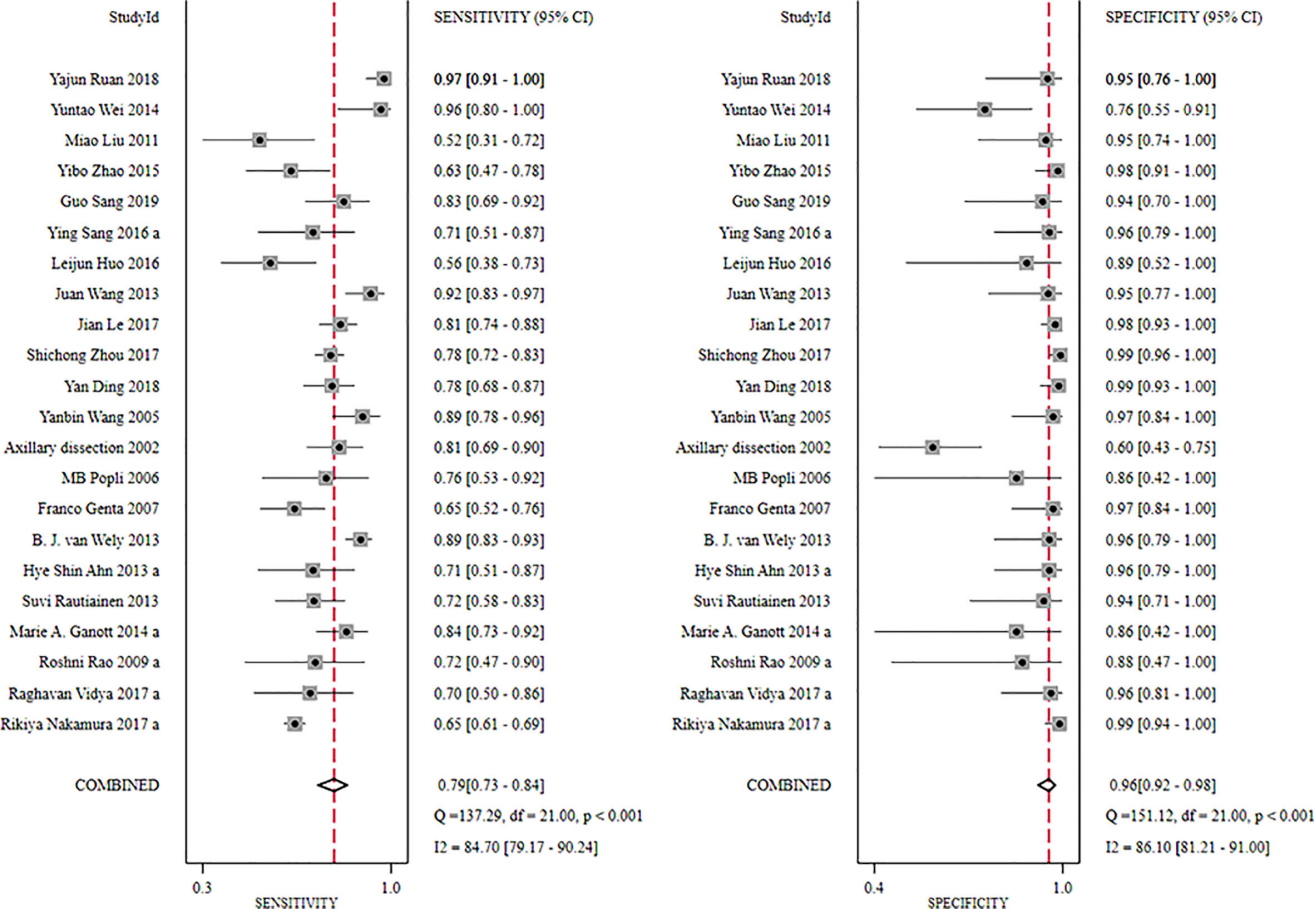
Forest plot showing the sensitivity and specificity values of US-FNA for suspicious axillary lymph nodes.

**Figure 3:**
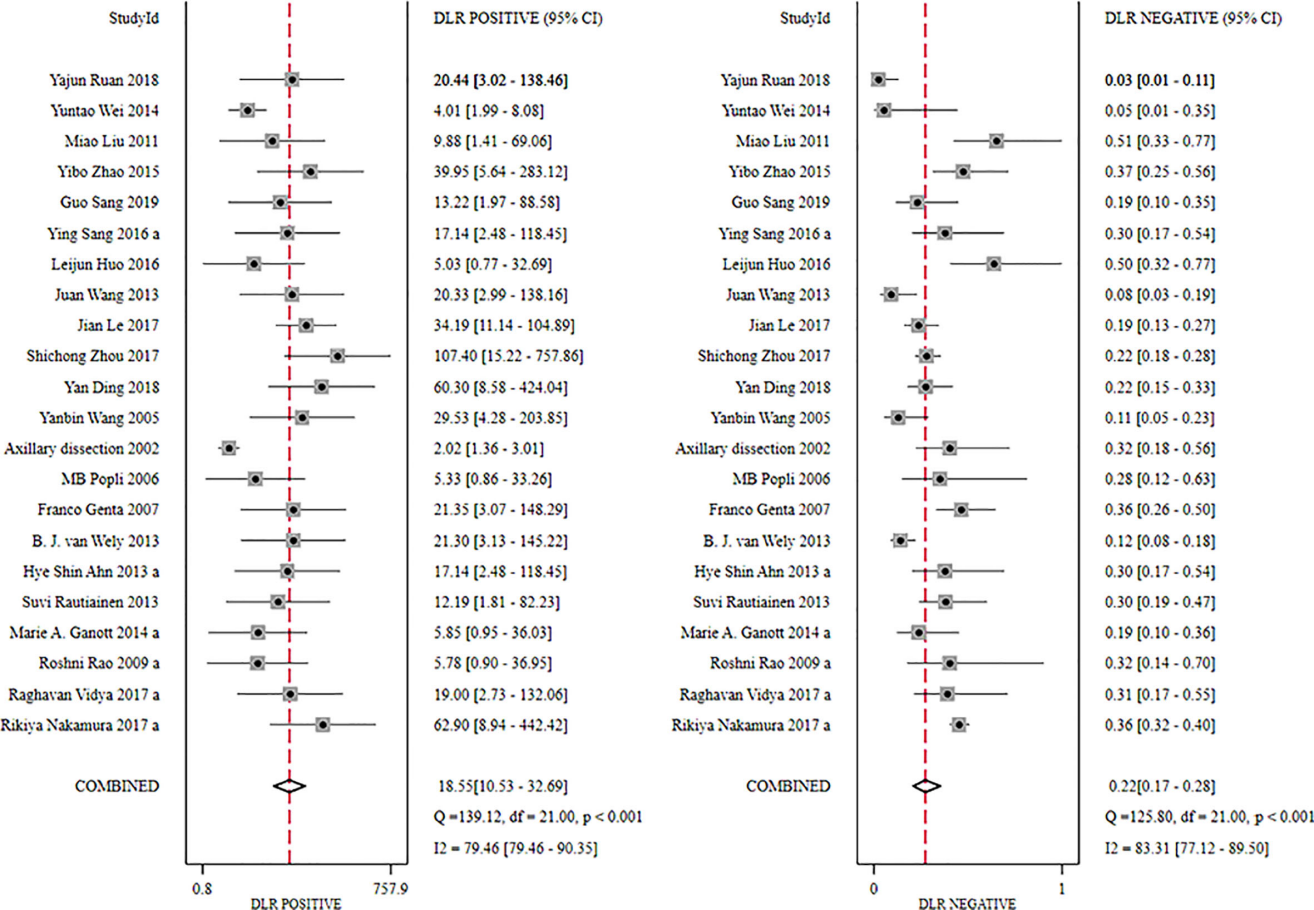
Forest plot showing the positive and negative likelihood ratio of US-FNA for suspicious axillary lymph nodes.

**Figure 4:**
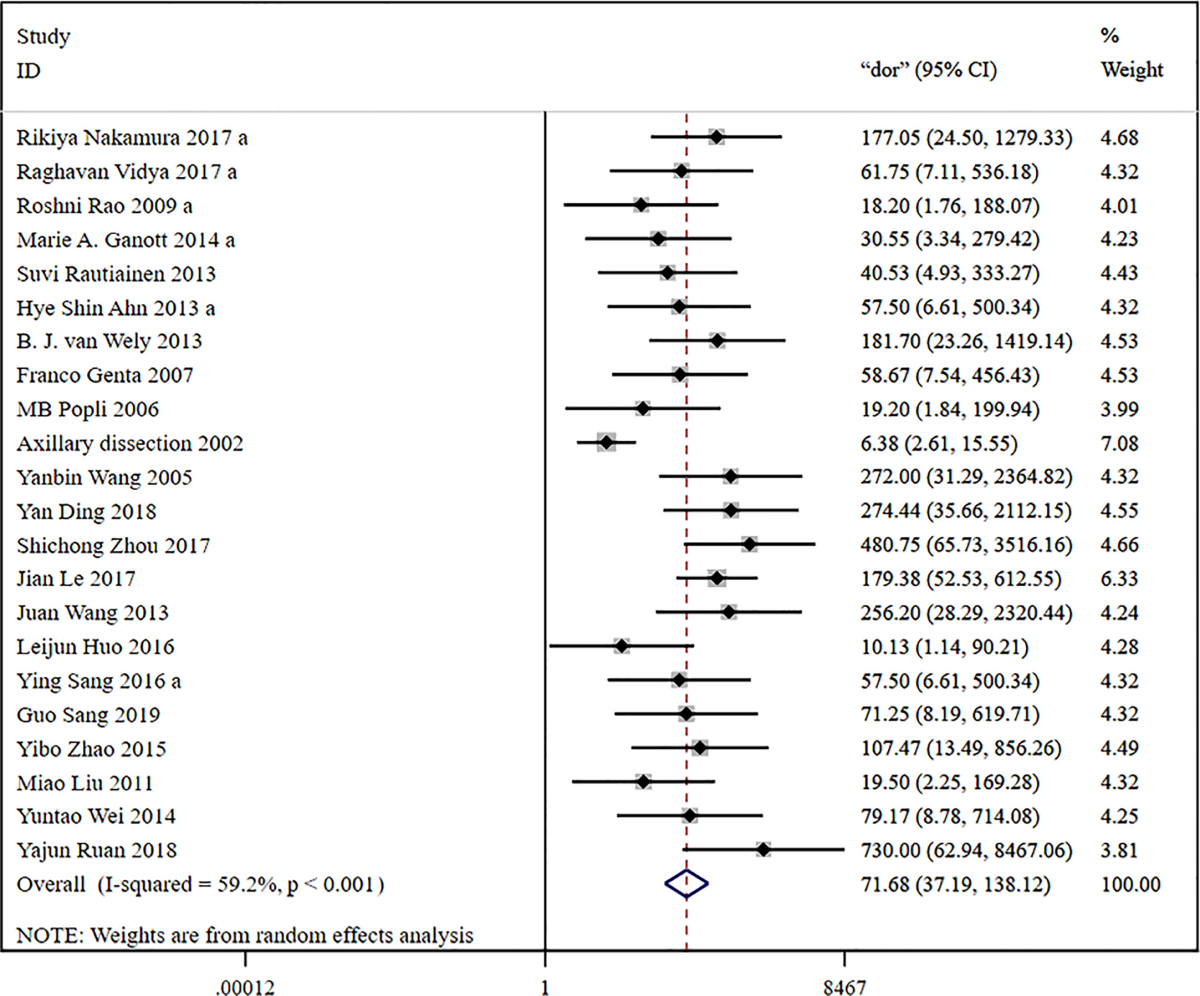
Forest plot showing the diagnostic odds ratio of US-FNA for suspicious axillary lymph nodes.

**Figure 5:**
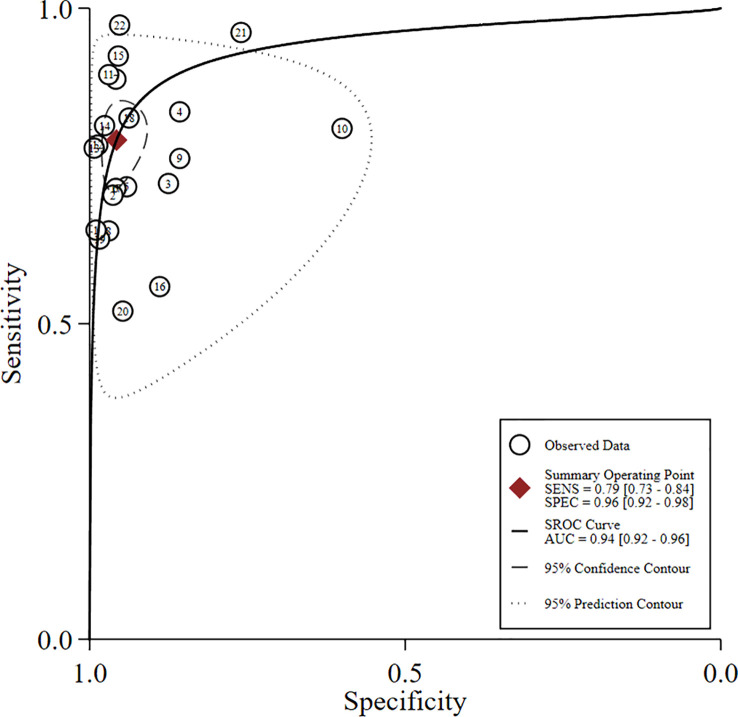
Summary ROC plot for diagnostic accuracy of US-FNA for suspicious axillary lymph nodes.

The global sensitivity was 79% (95% CI: 73%–84%), and the global specificity was 96% (95% CI: 92%–98%). The overall positive and negative likelihood ratios were 18.55 (95% CI: 10.53–32.69) and 0.22 (95% CI: 0.17–0.28), respectively, which suggested that US-FNA increased 18.6-fold the odds of the diagnosis of suspicious ALNs and decreased 0.22-fold the odds of the false-positive results. The overall DOR was 71.68 (95% CI: 37.19–138.12), which showed that the odds were 71.7-fold higher for the positive result among positive ALNs when compared with negative ALNs. The area under the SROC curve was 0.94 (95% CI: 0.92–0.96). All the data are shown in **Figures 2**–**5**.

**Figure 6:**
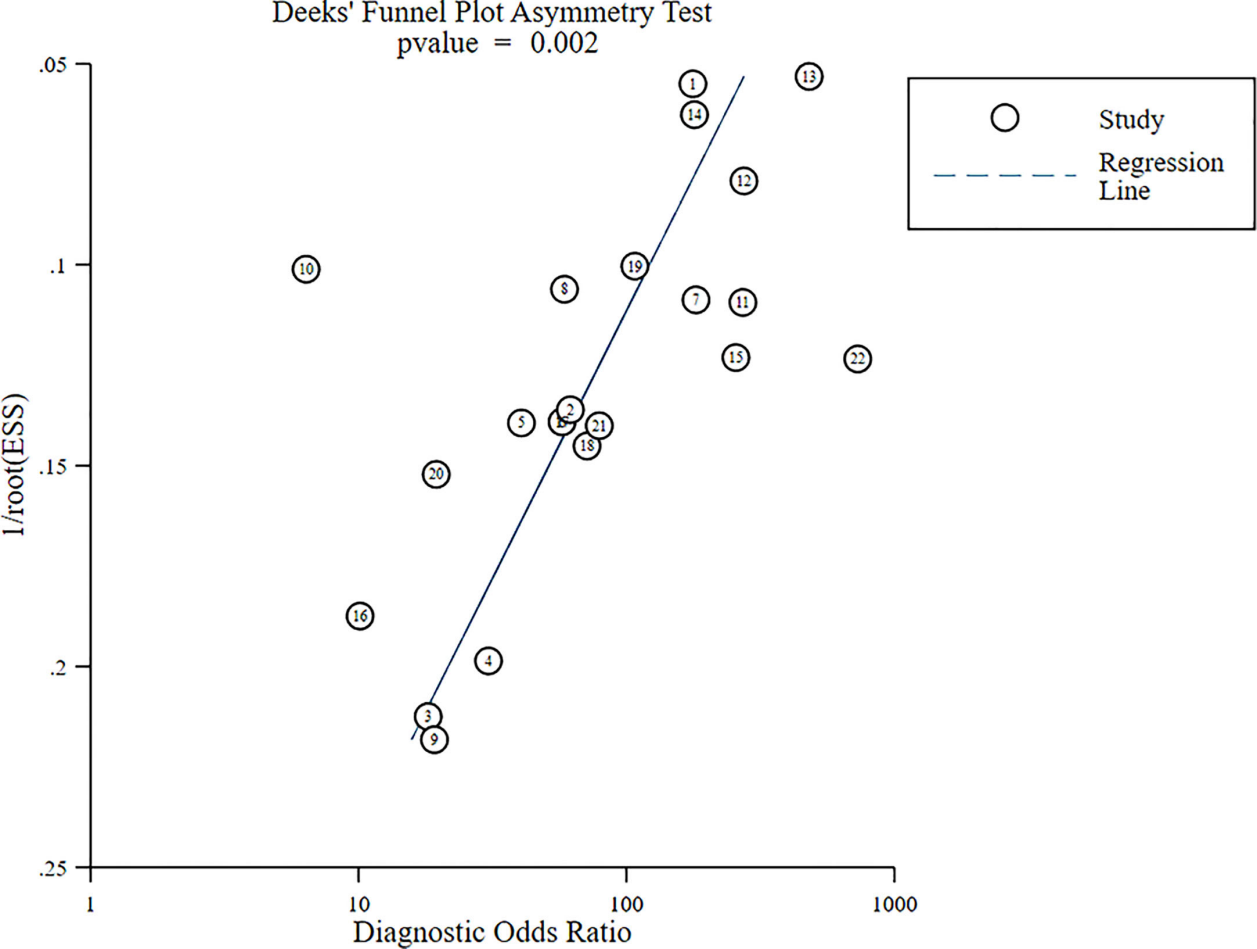
Funnel plot of US-FNA DOR meta-analysis.

The Deek’s funnel plot for DOR of US-FNA was asymmetric, indicating a significant publication bias (**Figure 6**, *p* = 0.002). The funnel plot revealed an apparent asymmetry, which suggested the presence of potential publication bias and inflated estimates due to methodological design flaws in small studies, and/or lack of publication of small trials with non-robust results.

### 3.3 Diagnostic accuracy of US-CNB

A total of 11 studies with 758 patients were included to estimate the accuracy of US-CNB in diagnosing ALNs in women with breast cancer. The fixed-effects model was selected for meta-analysis due to the absence of heterogeneity (*p* = 0.985, *I* ^2 = ^0.0%). The correlation result (Spearman’s *R* = −0.7963, *p* = 0.0034) between the logarithm of sensitivity and the logarithm of specificity indicated the presence of the effect of the threshold.

**Figure 7:**
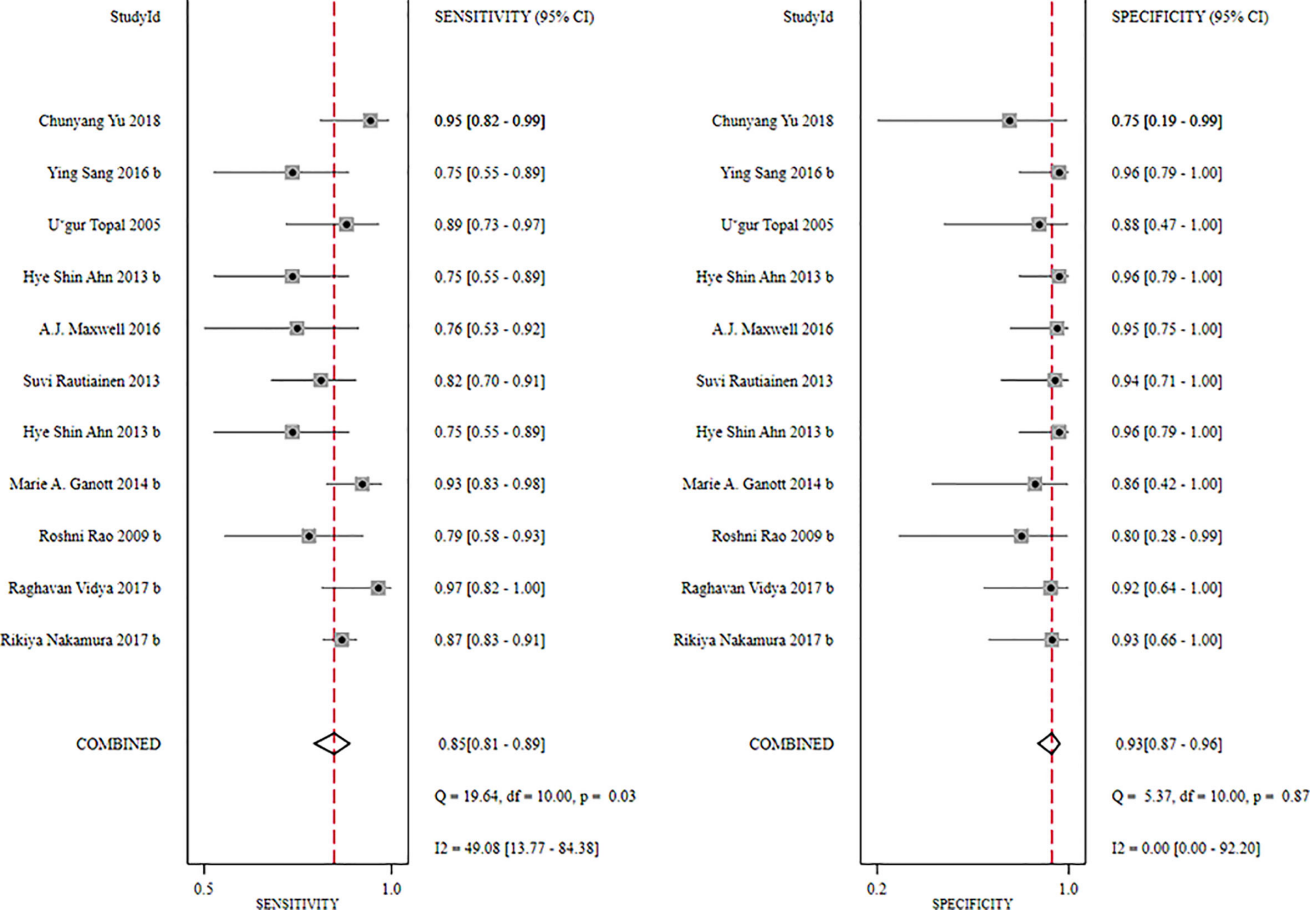
Forest plot showing the sensitivity and specificity values of US-CNB for suspicious axillary lymph nodes.

**Figure 8:**
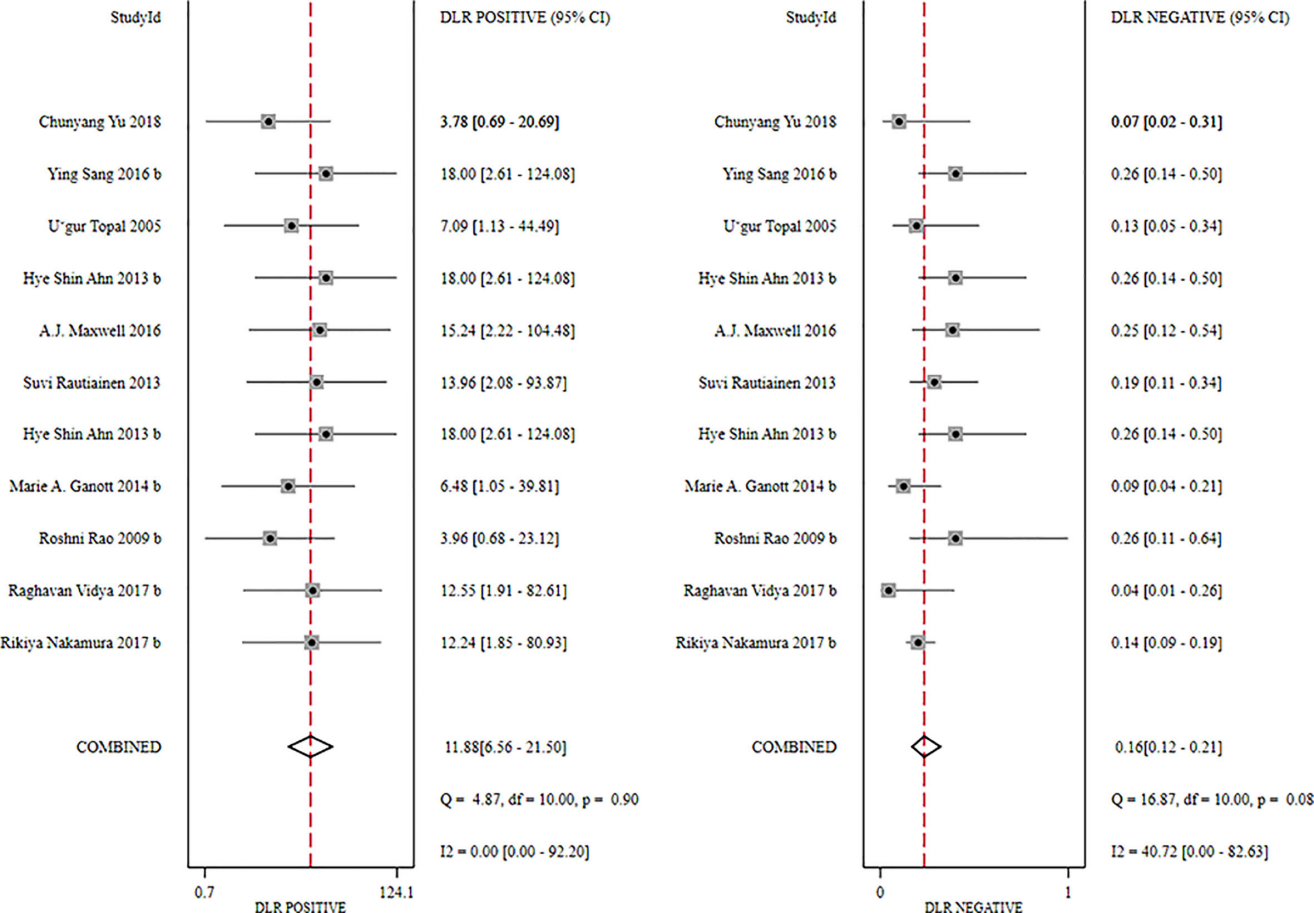
Forest plot showing the positive and negative likelihood ratios of US-CNB for suspicious axillary lymph nodes.

**Figure 9:**
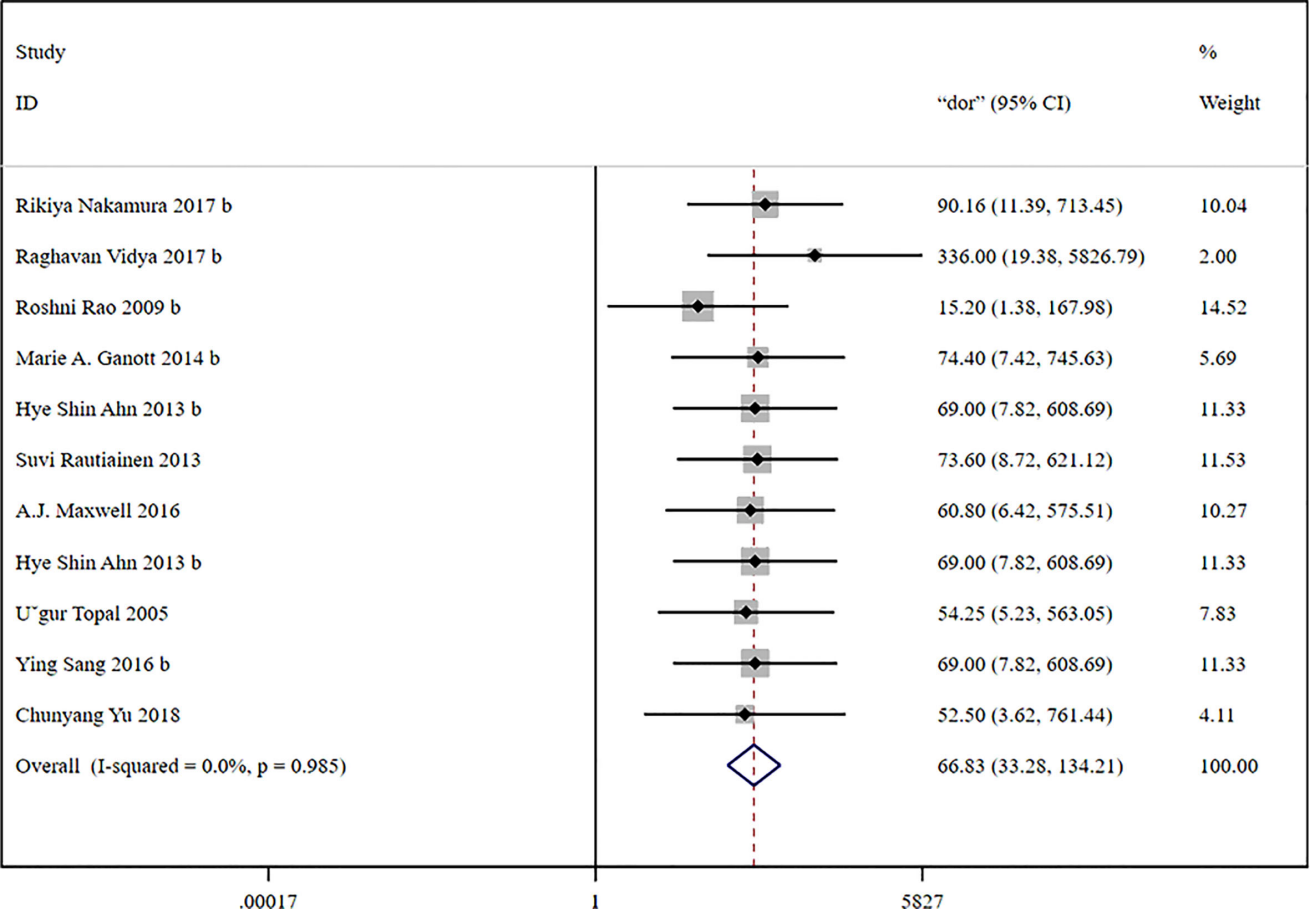
Forest plot showing the diagnostic odds ratio of US-CNB for suspicious axillary lymph nodes.

**Figure 10:**
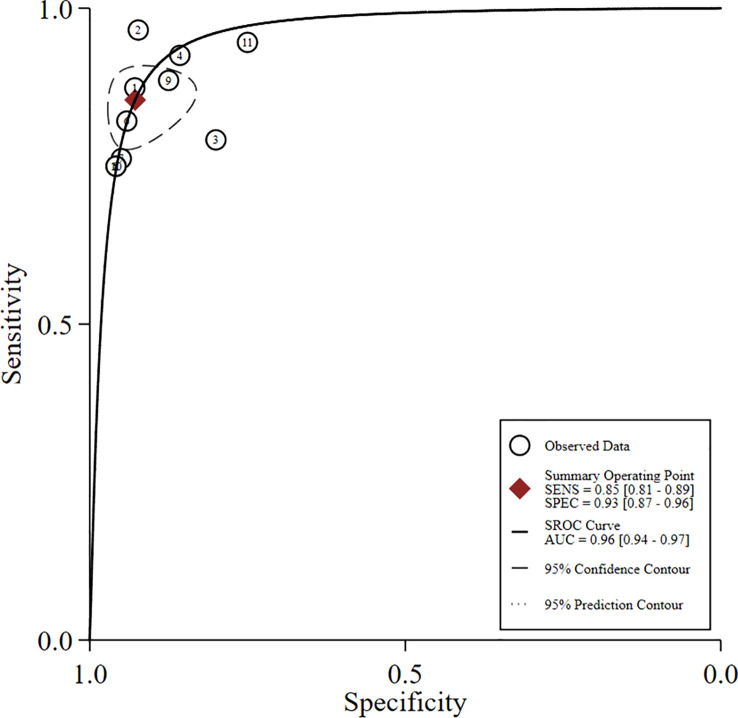
Summary ROC plot for diagnostic accuracy of US-CNB for suspicious axillary lymph nodes.

The global sensitivity was 85% (95% CI: 81%–89%), and the global specificity was 93% (95% CI: 87%–96%). The overall positive and negative likelihood ratios were 11.88 (95% CI: 6.56–21.50) and 0.16 (95% CI: 0.12–0.21), respectively. Thus, US-FNA increased 11.9-fold the odds of the diagnosis of suspicious ALNs and decreased 0.16-fold the odds of the false-positive result. The overall DOR was 66.83 (95% CI: 33.28–134.21), showing that the odds were 66.8-fold higher for the positive US-FNA result among positive ALNs when compared with negative ALNs. The area under the SROC curve was 0.96 (95% CI: 0.94–0.97), which indicated that the combined diagnosis was effective. All the data are shown in **Figures 7**–**10**.

**Figure 11:**
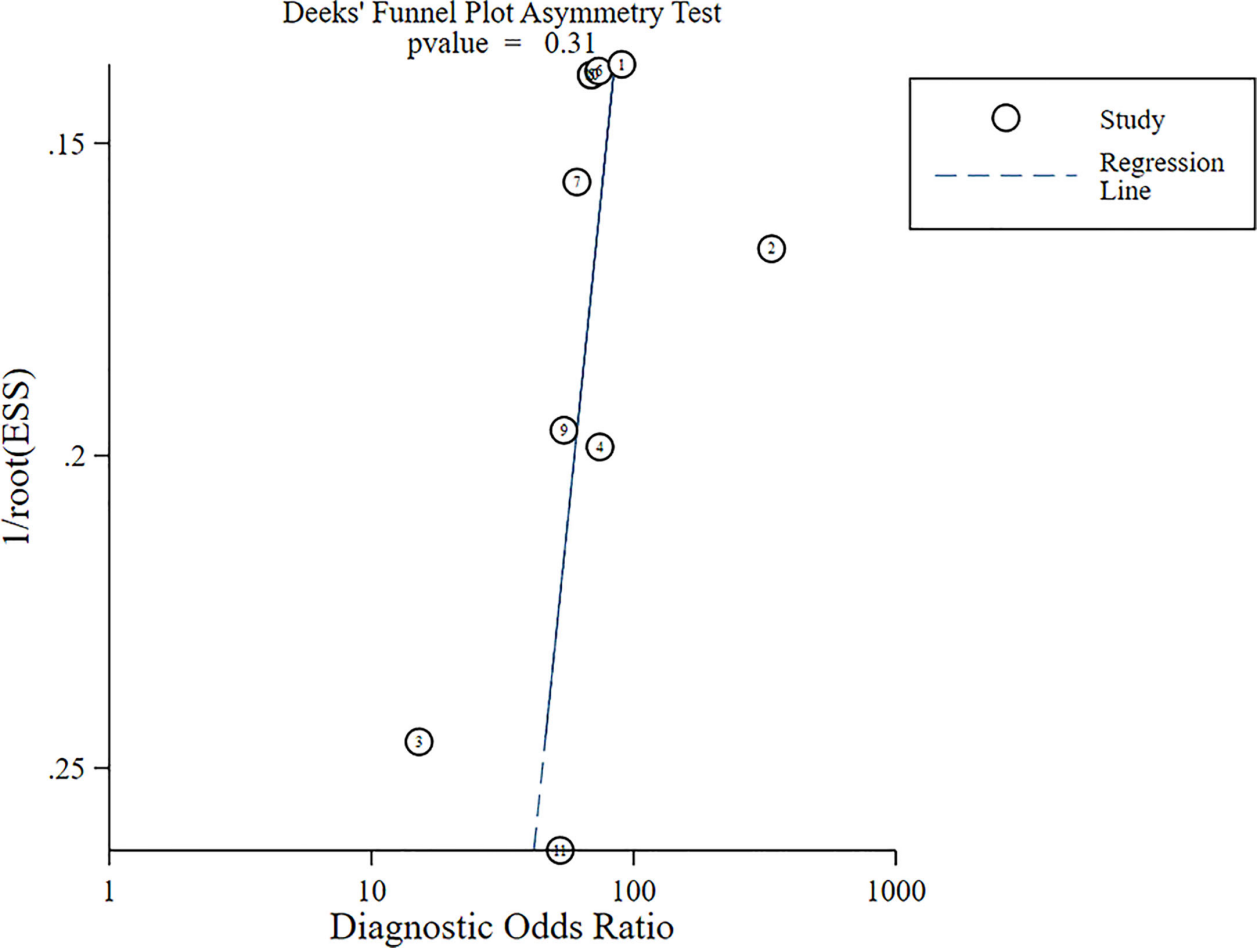
Deek’s funnel plot of US-CNB DOR meta-analysis.

The Deek’s funnel graph for DOR of US-CNB was symmetric, indicating no significant bias of publication (**Figure 11**, *p* = 0.31).

## 4 Discussion

This meta-analysis found that both US-FNA and US-CNB have high diagnostic accuracy in detecting ALN metastasis in patients with breast cancer. For detecting ALN metastasis, the sensitivity and specificity of US-FNA were 79% and 96%, whereas the sensitivity and specificity of US-CNB were 85% and 93%, respectively. The area under the SROC curve was 0.94 for US-FNA and 0.96 for US-CNB.

In the early stages of ALN metastasis of breast cancer, tumorous cells are first implanted in the marginal lymph node sinus by lymphatic infusion and then spread into the medullary sinus. At a later stage, the lymph node is completely occupied by the cancerous cells, and the cancer continues to develop. Cancer cells break through the capsule and adhere to the surrounding tissues, accompanied by the proliferation of the surrounding interstitial fibrous tissues, resulting in poor mobility of the lymph nodes, increase in stiffness, and less deformation by compression leading to an enlarged blue range in the elastogram.

Clinical palpation (PE), mammography (MMG), ultrasonography (US), computed tomography (CT), magnetic resonance imaging (MRI), positron emission tomography (PET), SLNB, FNA, and CNB are used to determine ALN status. PE examines superficial lymph nodes, but it is not meant for detecting metastasis as it has been found to be associated with considerably high false positives or false negatives and the reported sensitivity is 30% in detecting ALN metastasis in women with breast cancer (35). MMG can only detect the anterior ALNs and cannot completely cover the entire ALN area. MMG has a limited ability in distinguishing between benign tumors and malignancies in lymph nodes. In detecting metastasis in lymph nodes in patients with breast cancer, the sensitivity and specificity of MMG have been reported to be 21% and 80%, respectively (36).

CT is not much used in the detection of ALN metastasis in women with breast cancer. However, its use in estimating the extent of disease in advanced cases is more important (36). A sensitivity of 72% and a specificity of 40% of CT is reported in diagnosing ALN metastases in breast cancer patients who received neoadjuvant chemotherapy (37). MRI has a strong soft tissue resolution without radioactive damage, but the examination procedure is complicated, time-consuming, and expensive. MRI has no major role in the diagnosis of ALNs in breast cancer patients because of its limited ability to visualize the axilla although dedicated protocols have led to the attainment of high sensitivity (84%) and specificity (95%), which are not feasible in routine clinical practice (38). PET has a low spatial resolution and yields a considerable false-negative rate. The sensitivity of PET is low for smaller metastases and is unreliable for micro-metastases, and therefore, it is not usually recommended for ALN metastasis detection (39). The US is the preferential method used for the detection of ALN metastasis, which not only can help in visualizing the dimensions and contours of lymph nodes but also can detect the changes in cortical shape and texture indicative of the presence of metastasis (15, 38).

US-FNA is an efficient method of detecting lymph node metastasis in the axilla of breast cancer patients with high potential for predicting positive cases that can help in the initial staging of the tumor and deciding appropriate management strategies such as SLNB or ALND (15, 40). SLNB is a minimally invasive method with high diagnostic accuracy for ALN status determination that can be used to avoid ALND, which is associated with serious complications leading to a significant morbidity and compromised quality of life of patients (41–43). Histopathological examination after ALND is a reliable method for the diagnosis of ALN metastasis. However, ALND may cause many complications such as lymphatic reflux disorder, neuropathy, and shoulder stiffness in the affected upper arm after operation, thus affecting the upper limb function of patients. SLNB helps determine the nature of ALNs in breast cancer, and ALND is not required for negative SLNB results. The false negative rate of SLNB is about 5%–10%, with less trauma and fewer complications, but there may also be local effusion, sensory nerve injury, lymphedema, and other complications, and the occurrence of complications is closely related to the surgeon’s proficiency.

US-CNB of breast masses is a highly valuable technique. Any ultrasound-detected lesion can be subjected to CNB. US-CNB has several advantages including good needle control in real time, accessibility to problematic positions such as the axilla, acquisition of samples from multiple lesions, comfort to patients and radiologists, and cost-effectiveness (44). US-CNB has been found to yield better diagnostic accuracy than US-FNA in detecting axillary node metastasis in breast cancer patients (45). Based on the histological results from the puncture biopsy, the appropriate treatment for breast lesions can be determined. Under the guidance of ultrasound, CNB and FNA cytology play important roles in the diagnosis of breast lesions to improve the early diagnosis rate of breast cancer, which can improve the prognosis. FNA and CNB, which are the most widely used minimally invasive breast biopsy technologies, are mainly characterized by high accuracy, fast speed, small wound, few complications, and lower costs.

Neoadjuvant chemotherapy has been identified as a factor affecting the discrepancy between the initial and final staging of axillary nodes (26). A study found a significantly different rate of neoadjuvant chemotherapy between patients with false-negative results and those correctly diagnosed with percutaneous biopsy (21). Another study reported better sensitivity of US-CNB than US-FNA (92.5% vs 85%) in neoadjuvant chemotherapy-treated and (76.9% vs 65.4%) in no chemotherapy-treated patients. Patients treated with neoadjuvant chemotherapy had a higher level of abnormal appearance of nodes and had thicker cortices (12). A meta-analysis of 16 studies found 94% sensitivity and 6% false-negative rate of SLNB after neoadjuvant chemotherapy in staging axillary nodes in breast cancer patients who were node-negative initially (46) whereas another meta-analysis of 19 studies found 91% identification rate and 13% false-negative rate of SLNB after neoadjuvant chemotherapy in breast cancer patients who were node-positive initially (47).

There are certain limitations of the present study: (1) only English and Chinese articles were included due to logistic and technical difficulties resulting in a selection bias, which might have some influence on the overall outcomes; (2) there can be some impact of the variabilities in the operation of procedure on the outcomes of individual studies; (3) the presence of benign or non-cancerous lesions can also affect the results; and (4) a limited number of histological specimens may have influenced the accuracy of biopsy results of individual studies. However, the meta-analysis tends to moderate such effects by pooling the outcomes of several studies and entertaining their individual heterogeneities and thence minimizes the impacts of such factors. In our meta-analyses, statistical heterogeneity was not high, which may indicate the some influence of clinical and methodological heterogeneity; (5) only pooled data were analyzed, as individual patient data were not available, and this precluded more in-depth analyses.

## 5 Conclusion

Both US-CNB and US-FNA have good diagnostic accuracy in identifying suspicious ALNs in patients with breast cancer. With 85% sensitivity, 93% specificity, and the area under the SROC curve of 0.96, US-CNB appears to have better diagnostic efficiency than US-FNA, which is found to have 79% sensitivity, 96% specificity, and an area under the SROC curve value of 0.94.

## Data availability statement

The datasets presented in this study can be obtained from the corresponding author upon a reasonable request.

## Author contributions

Substantial contributions to conception and design: HZ, RZ, and WW. Data acquisition, data analysis, and interpretation: XL, XW, CW, and YR. Drafting the article or critically revising it for important intellectual content: HZ, RZ, and WW. Agreement to be accountable for all aspects of the work in ensuring that questions related to the accuracy or integrity of the work are appropriately investigated and resolved: All authors. All authors contributed to the article and approved the submitted version.
